# The risk of breast cancer in women with a CHEK2 mutation

**DOI:** 10.1186/1897-4287-10-S1-A5

**Published:** 2012-01-12

**Authors:** Cezary Cybulski, Dominika Wokołorczyk, Anna Jakubowska, Tomasz Huzarski, Tomasz Byrski, Jacek Gronwald, Tadeusz Dębniak, Bohdan Górski, Steven A Narod, Jan Lubiński

**Affiliations:** 1 International Hereditary Cancer Center, Department of Genetics and Pathology, Pomeranian Medical University, Szczecin, Poland

## 

Mutations in CHEK2 predispose to a range of cancer types including breast cancer. A meta-analysis of all association studies estimated the risk of breast cancer among carriers of 1100delC to be increased by 2.7-fold (9) and increased by 4.7-fold among carriers with a positive family history of breast cancer (Weischer M et al. CHEK2*1100delC genotyping for clinical assessment of breast cancer risk: meta-analysis of 26,000 patients cases and 27,000 controls. J Clin Oncol 2008; 26: 542-548). We estimated the risk of breast cancer in a woman who has a CHEK2 mutation depending on her family history of breast cancer. Out data suggest that carriers of a truncating mutation of CHEK2 (IVS2+1G>A, del5395, 1100delC) have 2.9 – fold increased risk of breast cancer in the Polish population. The risk was higher for women with at least one first-degree relative with breast cancer (OR = 4.5), and for women with at least one second-degree relative with breast cancer (OR = 3.5). If both a first- and second-degree relative was affected with breast cancer, the odds ratio was 6.4. We estimate the lifetime risks for carriers of CHEK2 truncating mutations to be from 21 to 37% depending family history of breast cancer in first- and second degree relatives. CHEK2 mutation screening detects a clinically meaningful risk of breast cancer.

